# Association of *Mouse Double Minute 2 -309T>G* Polymorphism with Acute Myeloid Leukemia in an Iranian Population: A Case- Control Study

**DOI:** 10.31557/APJCP.2019.20.10.3037

**Published:** 2019

**Authors:** Mona Soleymannejad, Mohammad Hassan Sheikhha, Hossein Neamatzadeh

**Affiliations:** 1 *Department of Biology, Ashkezar Branch, Islamic Azad University, *; 2 *Department of Medical Genetics,*; 3 *Mother and Newborn Health Research Center, Shahid Sadoughi University of Medical Sciences, Yazd, Iran. *

**Keywords:** Acute myeloid leukemia, mouse double minute 2, MDM2, 309T>G, polymorphism

## Abstract

**Background:**

Genetic factors play a substantial role in acute myeloid leukemia (AML) etiology. Overexpression of the *mouse* *double minute 2 (MDM2)* gene has been explored in many tumors. However, the role of *MDM2 -309T>G (rs2279744)* polymorphism in AML remains unclear. We have performed this study to examine the association of *MDM2 -309T>G *with AML in an Iranian population.

**Methods:**

We have examined the association of *N MDM2 -309T>G* polymorphism in 73 cases diagnosed with AML and 80 healthy controls by tetra-primer amplification refractory mutation system (ARMS) PCR assay. Odds ratios (OR) and 95% confidence intervals (CI) were calculated on the risk genotypes and alleles.

**Results:**

The TT, GG and GG genotypes of *MDM2 -309T>G *polymorphism in patients were 32.9%, 23.2% and 43.9%, while in controls were 86.2%, 7.5% and 6.3%, respectively. Moreover, Frequency of mutant allele (G) was 55.6% in cases with AML and 10.0% in controls. The mutant homozygote genotype (GG) was associated with an increased susceptibility to AML (OR 1.471; 95% CI: 1.062-1.844; p=0.004).

**Conclusion:**

Our results showed that the *MDM2 -309T>G* polymorphism was significantly associated with increased risk of AML in the Iranian population. Thus, the *MDM2 -309T>G* polymorphism might be useful genetic susceptibility factors in the pathogenesis of AML.

## Introduction

Acute myeloid leukemia (AML) is a severe and life threatening malignancy which occurs in all ages but is more common in the elderly with a median age at diagnosis of approximately 70 years (Almeida and Ramos, 2016; Deschler and Lübbert, 2006; Masoumi-Dehshiri et al., 2014; Nazha and Ravandi, 2014). AML is a clonal disease characterised by a variety of hematopoietic malignancies in the bone marrow, presenting impaired differentiation, deregulated proliferation and a survival advantage of myeloid progenitor cells (Corces et al., 2017; Khwaja et al., 2016). AML has an incidence of three to five cases per 100,000 population in the developed with a poor clinical outcome (De Kouchkovsky and Abdul-Hay, 2016; Kumar, 2011). In the United States, there are approximately 20,000 new cases of AML diagnosed per year, with death rate close to 50% (De Kouchkovsky and Abdul-Hay, 2016; Deschler and Lübbert, 2006). The etiology of AML is generally unknown (Löwenberg and Rowe, 2016).

It is clear that the environmental factors such as previous treatment with chemotherapeutic agents as well as preceding haematological malignancies, such as myeloproliferative disease and myelodysplastic syndromes increase the risk of later developing AML (Krishnan et al., 2000; Ramadan et al., 2012). Chromosomal rearrangements have been detected in almost half of AML cases (De Kouchkovsky and Abdul-Hay, 2016). A thorough examination of genetic variation is necessary for an exactly understanding etiology and development of AML. AML is also speculated to be associated with mutation in genes that plays an important role in tumours suppressor (Kamali et al., 2017). As well as, the *Mouse Double Minute 2 (MDM2)* gene is amplified and/or overexpressed in leukemia and constitutes one of a number of ways the p53 pathway is disrupted in development of these malignancies (Dimitriadi et al., 2008; Ebid et al., 2012).

The* MDM-2 (MIM#164785)* gene encodes a 90-kDa protein that binds specifically to the *p53* tumor suppressor and directly inhibiting the transcriptional activity of* P53 *(Ard et al., 2002; Dimitriadi et al., 2008; Falk et al., 2015). Moreover, *MDM2* functions as an E3 ubiquitin ligase is responsible for the ubiquitination and proteolytic degradation of p53 (Gohari et al., 2016; Neamatzadeh et al., 2015). It is known that *MDM2* has two promoters, a basal promoter (P1) and an alternative promoter (P2) starting in the intron 1. The P1 promoter is a constitutive promoter that does not affect the expression levels of *MDM2*. The P2 promoter is a *p53*-dependent intronic promoter containing two adjacent* p53* binding elements (Ard et al., 2002). P2 may affect the expression levels of *MDM2*. The human *MDM2* gene is mapped on chromosome *12q14.3-15* and contains 11 exons. The *MDM2* gene contains several polymorphisms, which *MDM2 -309T>G* polymorphism (*rs2279744*) is most studies SNPs in various tumors and located on the within the intronic p53-responsive promoter of the *MDM2 *(*P2*)(Chen et al., 2014; Hashemi et al., 2014). The *MDM2 -309T>G* polymorphism has been associated with the increased affinity of the transcriptional activator Sp1, resulting in higher levels of *MDM2 mRNA* and protein (Dimitriadi et al., 2008).

It has been showed that the *MDM2 -309T>G *polymorphism might be increased risk of AML. But the results are inconsistent and inconclusive. In order to get more precision results for the *MDM2 -309T>G* polymorphism and the risk of AML, we performed this case-control study among an Iranian population.

## Materials and Methods

*Participants*

This study was formally approved by the Ethics Committee of Ashkzar Universities of Medical Science and a written informed consent was obtained from each cases and controls. All procedures were performed in accordance with the approved guidelines. We recruited 73 patients diagnosed with de novo AML and 80 age and sex matched healthy controls between 2015 and 2016 from Shahid Sadoughi hospital at Yazd city. The diagnosis of AML was established according to the customary FAB criteria, and was based on bone marrow morphology and cytochemical staining.

The sample size in cases was estimate by using the following formula:

$n=\frac{z^{2}p(1-p)}{d^{2}}$

*Genotyping*

Genomic DNA was extracted from 0.2 ml fresh peripheral blood samples using a DNA kit (Cinnagen, Tehran, Iran) according to the manufacturer’s protocols and stored at −20°C until used for genotyping. Extracted DNA samples had a final concentration ranging from 100-200 ng/µL. The *MDM2 -309T>G* polymorphism was genotyped using tetra-primer amplification refractory mutation system (tetra-ARMS) PCR method. The primers sequences were shown in Table 1. The PCR reactions mixture contained approximately 50 ng of DNA, 1.5 μL TaqMan master mix (Applied Biosystems) containing Taq DNA polymerase and dNTPs (Applied Biosystems, Foster City, USA), 1 μL each primers and distilled water for a final volume of 10 μL. Thermal cycling conditions were as 95°C for 15 minutes, then 35 cycles of 95°C for 45 seconds, 57°C for 30 seconds, and 72°C for 1 seconds and final extension of 72°C for 10 minutes.

*Statistical analysis*

Statistical analysis was performed by using the using Statistical Package for Social Science (SPSS) program version 18. We used a Chi-square goodness-of-fit test to examine the adherence of the *MDM2 -309T>G* polymorphism to the Hardy-Weinberg Equilibrium (HWE) in healthy subjects. The allele and genotype frequencies of *MDM2 -309T>G* polymorphism between cases and controls were analyzed by using Chi-square (χ^2^) test. Odds ratios (OR) and 95% confidence intervals (CI) were calculated on the risk genotypes and alleles. P-values were two-tailed and a value of less than 0.05 was considered statistically significant.

## Results

The demographic and clinical characteristics of patients with AML and controls are shown in Table 2. No significant difference was observed in age and gender between patients with AML and controls (P>0.05).

The genotype frequencies of *MDM2 -309T>G* polymorphism are summarized in Table 3. We found that the observed genotype frequencies for *MDM2 -309T>G* polymorphism in the controls did not in agreement with the Hardy-Weinberg equilibrium (p≤0.001), and the Minor allele frequencies (MAF) in healthy subjects was 10%.

The TT, GG and GG genotypes of *MDM2 -309T>G* polymorphism in cases with AML were 32.9%, 23.2% and 43.9%, respectively. While, in the control group those genotypes frequencies were 86.2%, 7.5% and 6.3%, respectively. There was a significant difference in homozygote mutant genotype (GG) frequency between cases and controls. Analysis showed that the homozygote mutant genotype (GG) was significantly associated with increased risk of AML (OR 1.471; 95% CI: 1.062-1.844; p=0.004). Frequency of mutant allele (G) was 55.6% in AML and 10.0% in healthy controls. There was a significant differences between cases and controls regarding the mutant allele (G) frequency (p=0.001). Analysis showed that the mutant allele (G) was significantly associated with increased risk of AML (OR 1.578; 95% CI: 0.984-2.017; p=0.00.1; Table 3).

According to the French-American-British (FAB) classification: the AML-M2 was the predominant FAB subtype (24.6%) followed by M3 (20.5%), M1 and M4 (15.1% each), M6 (8.2%), M0 and M5 (6.8%), M5 (7.2%), and M7 (2.7%). However, there were no significant differences between GG, GT and TT genotypes of *MDM2 -309T>G* polymorphism according to the FAB classification in AML cases (Table 4).

## Discussion

*MDM2* binds to the transactivation domain of the p53 protein and inhibits* p53*’s transcriptional activity (He et al., 2015). *MDM2* overexpression can cause dysfunction and inactivation of TP53, which promotes tumor progression. Since the first publication on *MDM2 -309T>G* polymorphism in 2004, several studies have been evaluated the association of *MDM2 -309T>G* polymorphism with development of various malignancies (Bond et al., 2004). To date, a few studies have been evaluated the association *MDM2 -309T>G* polymorphism with AML risk (He et al., 2015; Ou, 2015; Xiong et al., 2009). However, those studies results about association of *MDM2 -309T>G* polymorphism with development of AML are inconclusive.

**Table 1:** The Primers for MDM2 -309T>G Polymorphism (rs2279744) Identification

Primers Name	Sequence
T Allele Forward	3’-GGGGGCCGGGGGCTGCGGGGCCGTTT-5’
T Allele Reverse	3’-TGCCCACTGAACCGGCCCAATCCCGCCCAG-5’
G Allele Forward	3’-GGCAGTCGCCGCCAGGGAGGAGGGCGG-5’
G Allele Reverse	3’-ACCTGCGATCATCCGGACCTCCCGCGCTGC-5’

**Table 2:** Characteristics of the Children with AML and Controls

Variable	AML (n=73)	Controls (n=80)	P-value
Gender			
Male	41 (56.2)	40 (50.0)	0.121
Female	32 (43.8)	40 (50.0)	
Age			
18-35	15 (20.5)	20 (25.0)	
36-50	27 (37.0)	27 (33.7)	0.125
51-78	31 (42.5)	33 (41.3)	

**Table 3:** Genotypes and Alleles Frequency of the MDM2 -309T>G Polymorphism in AML and Controls

Polymorphism	AML (n=73)	Controls (n=80)	OR	95% CI	P-value
Genotype					
TT	24(32.9)	69(86.2)	Ref.		
TG	17(23.2)	6(7.5)	1.213	0.885-1.986	0.05
GG	32(43.9)	5(6.3)	1.471	1.062-1.845	0.004
Allele					
T	65(44.5)	144(90.0)	Ref.		
G	81(55.6)	16(10.0)	1.578	0.984-2.017	0.001

**Table 4:** Distribution of MDM2 -309T>G Polymorphism Genotypes in AML Patients According to the FAB Classification

FAB	Homozygote Wild TT (n=24)	Heterozygote TG (n=17)	Homozygote Mutant GG (n=32)	P-Value
M0	2 (8.3)	1 (5.9)	3 (9.4)	0.81
M1	3 (12.5)	4 (23.5)	4 (12.5)	0.51
M2	6 (25.0)	5 (29.5)	7 (21.9)	0.76
M3	6 (25.0)	3 (17.6)	6 (18.7)	0.67
M4	3 (12.5)	2 (11.7)	6 (18.7)	0.41
M5	1 (4.1)	1 (5.9)	3 (9.4)	0.21
M6	2 (8.3)	1 (5.9)	2 (6.2)	0.24
M7	1 (4.1)	0 (0.0)	1 (3.1)	0.83

In the current study we have evaluated the association of *MDM2 -309T>G* polymorphism with AML risk in an Iranian population. To our best knowledge, this is the first study on the relationship of *MDM2 -309T>G* polymorphism with AML risk in Iranian and also Middle East populations. Our results showed that the *MDM2 -309T>G* polymorphism was significantly associated with increased risk of AML in the Iranian population. The biological mechanisms of this susceptibility may be as follows: the G allele of *MDM2 -309T>G* polymorphism could significantly increase the affinity of *MDM2* to transcriptional activator SPl, resulting in increased mRNA transcription and protein expression of *MDM2*. Thus, *MDM2* directly inhibits the *p53* tumor suppressor pathway and increasing risk of AML in people with mutant allele (G).

In 2009, Xiong et al., (2009) for first have examined the association of *MDM2-309T>G* polymorphism and *p53* codon 72 polymorphisms with risk of AML in a Chinese population. They have revealed that the mutant allele (G) of *MDM2-309T>G* polymorphism was associated with increased risk of AML. However, their results failed to show the association of *MDM2-309T>G* polymorphism and *p53* codon 72 polymorphisms with age of onset and other clinical parameters of patients. In 2015, Cingeetham et al., in case-control study of 223 de novo AML cases and 304 age and sex matched healthy controls have evaluated the association of *MDM2-309T>G* polymorphism with susceptibility to AML by tetra-primer amplification refractory mutation system (ARMS)-PCR method in an Indian population. There results showed that the *MDM2-309T>G* polymorphism was significantly associated with increased risk of AML in the Indian population. Moreover, their results revealed that the *MDM2-309T>G* polymorphism might be serve as a good prognostic indicator for AML (Cingeetham et al., 2015). In the same year, Falk et al., (2015) have examined the frequency and impact of *TP53* and *MDM2-309T>G* polymorphism on treatment outcome and overall survival (OS) in 189 AML patients in Swedish people. They have found that the *MDM2-309T>G *polymorphism might be useful for prognostication, risk stratification, and selection of AML cases most likely to benefit from new drugs targeting the p53 signaling pathway. Ebid et al., (2012) have performed a study to evaluate the prevalence of *P53*,* P21Ser31Arg* and *MDM2-309T>G* polymorphism s in AML and to compare it to apparently normal healthy controls for assessment of impact on risk in an Egyptian population. The study results supported that these genes polymorphism, especially the combination of *21Ser31Arg/ MDM2-309T>G* polymorphisms might be genetic susceptibility to AML. According to their results, the G allele of *MDM2-309T>G* polymorphism was itself without affect. However, it showed a synergistic effect with *P21 Ser/Arg *polymorphism in development of AML. Additionally, they have found that the* P53 Arg72Pro* polymorphism was not significantly associated with increased risk of AML among the Egyptian population. In 2010, Barnette et al., (2004) have assessed the relationship of *MDM2-309T>G* polymorphism with AML risk among 575 de novo acute cases treated on three Children’s Oncology Group protocols in USA. The study showed that the *MDM2-309T>G* polymorphism was associated with increased risk of pediatric AML, but does not impact overall response to therapy.

Ou et al., (2015) in a meta-analysis of ten case-control studies with 1889 cases and 5707 controls have evaluated the association between *MDM2-309T>G* polymorphism and susceptibility to leukemia. Their results showed that individuals carrying the mutant allele (G) had increased susceptibility to leukemia compared to individuals who carrying the wild allele (T) (OR=1.24, 95% CI 1.06, 1.45, P=0.007). Moreover, they have showed that the individuals with the homozygous mutant genotype (GG) had 1.47 times the risk of leukemia compared to individuals with the wild homozygote (TT) and heterozygote (TG) genotypes. In another meta-analysis, He et al., (2015) have the association of *MDM2-309T>G* polymorphism with prognosis of leukemia. In the meta-analysis, they have selected eleven studies with a total of 2,478 patients accrued. Their pooled results showed that cases with homozygote mutant genotype (GG) had a significantly increased risk of developing leukemia (HR 1.90, 95% CI 1.56-2.31, p ≤ 0.001). However, they have found that patients with heterozygote (GT) not significantly associated with increased risk of leukemia (HR 1.18, 95% CI 0.96-1.45, p = 0.11). In addition, their pooled data showed that the *MDM2-309T>G* polymorphism did not significantly associated with patient survival (HR: 1.29, 95% CI 0.79-2.13, p = 0.31).

In summary, the current study results support the association of *MDM2 -309T>G* polymorphism with increased risk of AML in the Iranian population. Thus, the *MDM2 -309T>G* polymorphism might be useful genetic susceptibility factors in the pathogenesis of AML. However, our results are still needed to obtain more precise results by further well-designed case-control studies with larger sample sizes and in different ethnicities.
